# Management of Transverse Vaginal Septum by Vaginoscopic Resection: Hymen Conservative Technique

**DOI:** 10.1055/s-0038-1670714

**Published:** 2018-10

**Authors:** Gennaro Scutiero, Pantaleo Greco, Piergiorgio Iannone, Giulia Bernardi, Francesca Greco, Luigi Nappi

**Affiliations:** 1 Department of Morphology, Surgery and Experimental Medicine, Section of Obstetrics and Gynecology, University of Ferrara, Azienda Ospedaliero-Universitaria S. Anna, Cona, Ferrara, Italy; 2 Department of Medical and Surgical Sciences, Institute of Obstetrics and Gynecology, University of Foggia, Foggia, Italy

**Keywords:** transverse vaginal septum, hysteroscopy, hematocolpos, laparoscopy, vaginal anomaly, septo vaginal transverso, histeroscopia, hematocolpos, laparoscopia, anomalia vaginal

## Abstract

Transverse vaginal septum is a rare female genital tract anomaly, and little is described about its surgical treatment. We report the case of a patient who wished to preserve hymenal integrity due to social and cultural beliefs. We performed a vaginoscopic resection of the septum under laparoscopic view, followed by the introduction of a Foley catheter in the vagina, thus preserving the hymen. After 12 months of follow-up, no septal closure was present, and the menstrual flow was effective. Vaginoscopic hysteroscopy is an effective method of vaginal septum resection, even in cases in which hymenal integrity must be preserved due to social and cultural beliefs.

## Resumo

Septo vaginal transverso é uma anomalia rara do trato genital feminino, e pouco é descrito sobre o tratamento cirúrgico. Relatamos o caso de uma paciente que desejava preservar a integridade do hímen devido a crenças sociais e culturais. Realizamos ressecção vaginoscópica do septo sob visão laparoscópica, seguida da introdução de um cateter de Foley na vagina, preservando assim o hímen. Após 12 meses de acompanhamento, não havia fechamento do septo, e o fluxo menstrual era eficaz. A histeroscopia vaginoscópica é um método eficaz de ressecção dos septos vaginais, incluindo os casos em que a integridade do hímen deve ser mantida devido a crenças sociais e culturais.

## Introduction

Transverse vaginal septum (TVS) is an uncommon anomaly of the female genital tract whose incidence ranges from 1:2100 to 1:84000.^1^ ^2^ ^3^

The etiology of this embryologic anomaly is still unknown; this anatomical condition is linked to autosomal recessive transmission, but, in most cases, a genetic origin cannot be found.^4^

Physiologically, the sinovaginal bulbs invaginate from the urogenital sinus and meet the Müllerian tubercle on the caudal end of the Müllerian ducts to form the vaginal plate that is then canalized to form the lower part of the vagina.^5^

Transverse vaginal septa are thought to result from a failure in the canalization of the vaginal plate at the point where the urogenital sinus meets the Müllerian duct.^1^

Genitourinary and gastrointestinal tract anomalies might be associated with TVS, such as: imperforate anus, mal-rotation of the gut, ectopic ureter with hypoplastic kidney, hydronephrosis, vesicovaginal fistula, and bicornuate uterus.^6^ ^7^ Rare defects include musculoskeletal defects, aorta coarctation and atrial septal defect.^7^

Transverse vaginal septum can develop anywhere in the vagina, and its more common locations are the upper part of the vagina, the junction between the vaginal plate and the caudal end of the fused Müllerian ducts.^1^ ^2^ Rock et al^7^ observed that 46% of septa were located in the upper vagina, 35%, in the middle, and 19%, in the lower vagina.

Imperforate septa usually present during adolescence with symptoms such as cyclical abdominal pain, obstructed menstruation, hematocolpos, and, occasionally, urinary retention.^1^ Hematocolpos is defined as a distended vagina filled with menstrual blood; hematometra is a term used to describe the distended uterus filled with blood caused by extreme accumulation of menstrual products.^8^

The diagnosis is suspected when an abdominal or pelvic mass is palpated, or when a foreshortened vagina is encountered and the cervix cannot be identified.^1^ Clinical examination, ultrasound, and magnetic resonance imaging (MRI) are all used in the diagnosis, and the MRI is useful before the surgery to determine the thickness and depth of the septum.^9^ ^10^

A review of the literature suggests that ultrasound is still the primary diagnostic tool used, while the MRI is particularly useful to delineate more complex gynecologic anatomies.^11^ ^12^

In the literature, several approaches are described, but none seems to be better than the others.^1^

It is deeply important to recognize as early as possible the disease to prevent patient complications, including endometriosis, hydronephrosis, possible pyelonephritis and superinfection that will cause sepsis. Moreover, the surgery should be performed by an expert and educated team with the goal of restoring the regular anatomy and physiology of the patient.^13^

We present a case of transverse vaginal septum in which the patient and her family expressed the strong desire to preserve hymenal integrity due to a sociocultural belief. We propose the management of the septum with vaginoscopic resection using a hysteroscope, under laparoscopic control, followed by the introduction of a Foley catheter in the vagina.

## Case Description

A 14-year-old virgin girl presented to the emergency department complaining of severe hypogastric pain. She presented suprapubic fullness, but did not present abdominal distension or other symptoms.

Despite the presence of secondary sexual features, she had never had menarche, and had a history of cyclical pelvic pain.

**Fig. 1:**
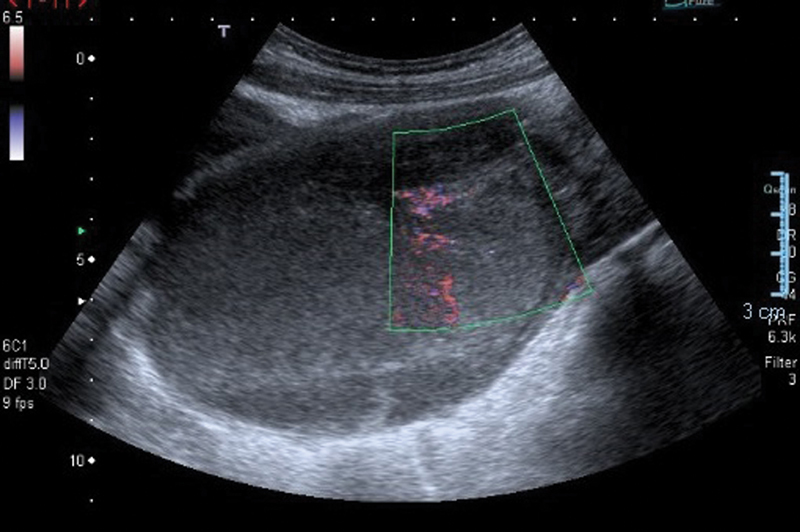
Abdominal ultrasound showing a distended uterus.

Upon examination, she had a tender suprapubic mass, palpable almost 2 cm under the umbilicus. A rectal examination revealed an anterior mass. An abdominal ultrasound revealed a distended uterus and a vagina filled with a homogenous thick fluid; a diagnosis of hematocolpometra was made (Fig. 1).

**Fig. 2:**
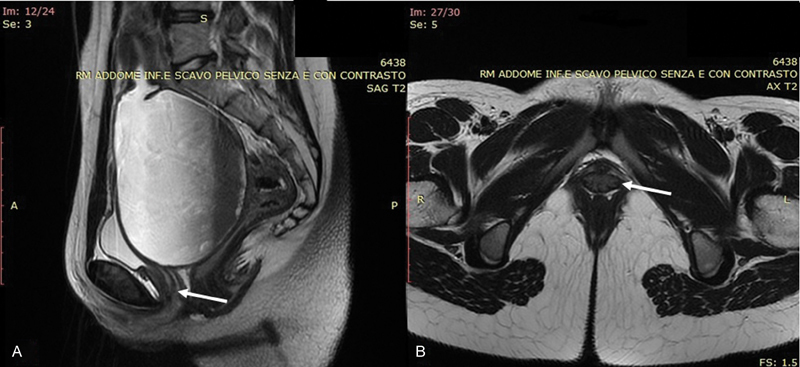
(A) Sagittal view of the magnetic resonance imaging scan: hematocolpometra and level of the septum (arrow); (B) transversal view of the septum (arrow).

An MRI was performed, and it showed proximal vagina and uterine distension, especially involving the cervix. The vagina appeared to terminate in a low complete transverse septum, ∼ 3 cm from the vaginal introitus, with a 7-mm thickness. No other abnormalities were detected (Fig. 2).

The patient and her family refused vaginal surgery to preserve hymen integrity due to a sociocultural belief. After counselling about performing the vaginoscopic resection of the septum using a hysteroscope under laparoscopic control, the patient and her family provided written informed consent for the procedure.

During laparoscopy, the uterus looked anteverted and enlarged. Both ovaries were normal. No endometriosis was detected.

The vagina appeared grossly distended, obliterating the Douglas pouch. A 5-mm office hysteroscope (Bettocchi Office Hysteroscope, Karl Storz GmbH & Co., Tuttlingen, Germany) was introduced through the hymenal opening according to the vaginoscopic approach technique.^14^ ^15^ The vaginoscopy revealed a bulging complete TVS. A 5-F bipolar electrode (Versapoint Bipolar Electrosurgical System, Gynecare, Ethicon, Inc., Somerville, NJ) was introduced through the operative channel of the hysteroscope, a bipolar electroresection of the septum was performed, and 300 mL of dark, old blood was evacuated. A Foley catheter was introduced from the introitus toward the septal perforation, and the balloon of the catheter was insufflated with 15 ml of fluid. The patient recovered well from the surgery, and was placed on continuous oral contraceptive pills to suppress her menses and prevent hematocolpos reaccumulation.

**Fig. 3:**
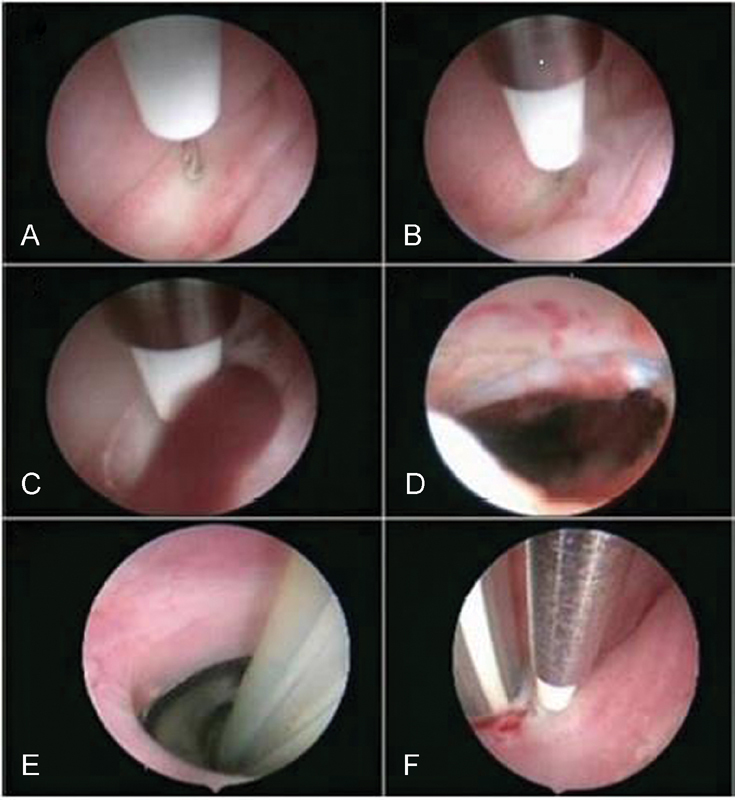
(A-B-C-D) 5-F bipolar electrode introduced in the operative channel of the hysteroscope and bipolar electroresection of the septum performed with dark old blood evacuated; (E) Foley catheter introduced from the introitus toward the septal perforation and the balloon of the catheter insufflated with 15 ml of fluid; (F) fibrotic ring resection with bipolar electrode without damage to the hymen.

She was dismissed three days after the procedure; a vaginoscopy was scheduled two months later, when the Foley catheter was removed. At the vaginoscopic examination, a fibrotic ring narrowing the vaginal canal was detected and completely resected with the bipolar electrode, without hymenal damage (Fig. 3).

**Fig. 4:**
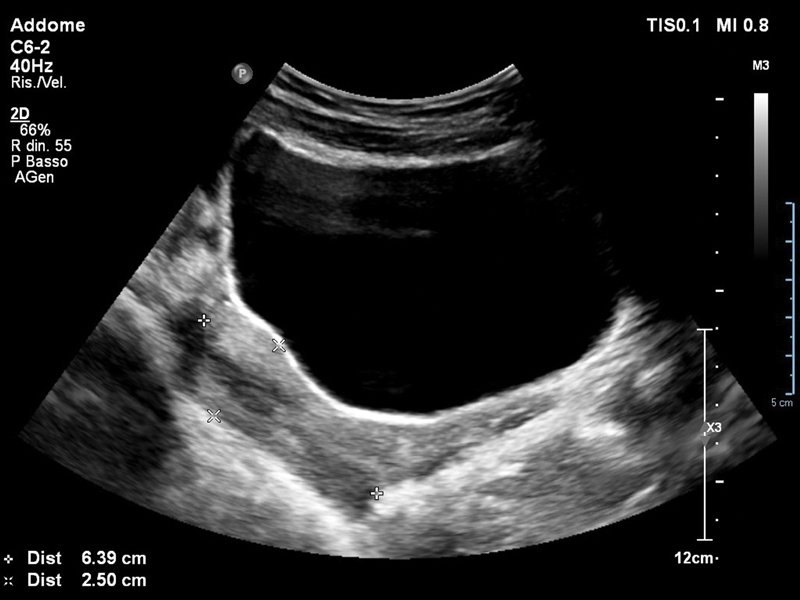
Normal uterus on ultrasound after 12 months.

The patient was discharged on the same day, and underwent regular follow-up. The oral contraceptive pill was stopped four months later. The patient experienced regular menses and no other symptoms or discomfort 6 and 12 months after the last procedure. An abdominal ultrasound scan showed a normal genital tract (Fig. 4).

## Discussion

One of the main causes of hematocolpometra is a complete vaginal septum.^16^ A transverse vaginal septum results from either incomplete canalization of the vaginal plate or failure of the paramesonephric ducts to meet the urogenital sinus.^7^ ^17^

Very few data are available in the literature about the surgical management. The studies report on septum location, thickness and perforation.^1^ ^3^ ^7^

The location is also based on the distance from the vaginal introitus to the distal end of the septum, and it can be low (< 3 cm), mid (3–6 cm), or high (> 6 cm). About the thickness, the septum can be thin (< 1 cm) or thick (> 1 cm). In the presence of perforation, the thickness can usually be determined upon vaginal examination. In cases of imperforate septum, the thickness of the septum is measured via MRI.^1^

According to Dennie's et al^18^ experience, the septum has to be removed when the girl reaches the menarche age, and the operative intervention is easier if the patient presents with hematocolpos before its drainage. Williams et al^1^ described 46 patients affected by TVS treated in 3 different ways: abdominoperineal vaginoplasty via laparotomy, simple excision using the vaginal approach, and laparoscopic resection of the vaginal septum.

Another surgical approach is the Grünberger method, which consists of a cross-shaped incision on the caudal part of the septum, a cruciate incision on the cranial part, and transverse closure.^19^ ^20^ Wierrani et al^21^ described good results in 13 patients treated with Grünberger modification of the Garcia Z plasty.

Van Bijsterveldt et al^22^ proposed two novel techniques for the treatment of the vaginal septum: the push through and pull through techniques. The first one requires a combined abdominal-vaginal approach, and it is used in patients presenting higher restenosis risk after surgery. The pull through technique is reserved for patients with a simple vaginal obstruction.^22^

A modification of this technique was performed by Layman et al^23^ with a pull through of a proximal distended vagina using an Olbert balloon catheter to facilitate the surgical management and to limit the postoperative narrowing of the vagina.

Vaginal septum may not be the only anatomical defect in a patient, but part of a more complex syndrome. Tug et al^24^ reported the case of a young patient affected by obstructed hemivagina and ipsilateral renal anomaly (OHVIRA) syndrome. In this case, the vaginal septum is completely incised by CO2 laser with hysteroscopy without hymenotomy, and the distal part of the septum is simply cut and sutured.^24^ Tehrani et al,^25^ also reported a single case of septum removal in a 19-year-old girl by resectoscope followed by hymenorraphy.

Sardesai et al^2^ described double cross plasty/Z plasty for the management of TVS after a 20-year experience as a better technique compared with the other surgical methods.

Several surgical techniques have been described, but Kansagra et al^26^ proposed an alternative approach. A method of serial balloon dilation over a transvaginally inserted guide wire to create a durable outflow tract from the uterus to the lower vagina, without the immediate need of surgery.^26^

Postoperative complications, such as vaginal stenosis and reobstruction, can occur, especially when the septum is thick.^3^ To decrease the chance of stenosis, vaginal molds can be used; however these require the cooperation of the young patient, who may not be emotionally mature enough to use dilators faithfully.^7^

In our case, since the patient and her family rejected any vaginal approach to preserve hymenal integrity, which represents virginity according to the sociocultural values and traditions of their society, we developed an alternative method that would not damage the hymen.

The vaginoscopic approach allowed us to preserve the integrity of the hymen. The risk of stenosis may still be present, but the pressure applied by the balloon of the catheter on the septal perforation is expected to decrease the closure of the septum. After 12 months of follow-up, there was no septal closure, and the menstrual outflow was effective. The patient did not complain of other symptoms or of discomfort after 12 months.

## Conclusion

In selected cases, with an intact outflow tract, vaginoscopy using a hysteroscope followed by the insertion of a Foley catheter could represent an effective method for the resection of vaginal septa, even in the case of virgin patients, because it is safe, effective, and easy to perform. The vaginoscopic approach should be used when performing hysteroscopy, but, above all, it should be considered as an alternative treatment in societies composed of ethnic groups that place importance on the integrity of the hymen.
